# Severe hyponatremia with seizures and confirmed mild brain edema by hysteroscopic myomectomy: a case report

**DOI:** 10.1186/s40981-020-00381-0

**Published:** 2020-10-01

**Authors:** Haruko Okazaki, Norikazu Miura, Yuki Kashima, Ryoichi Miyashita, Katsunori Oe, Keiko Kawakami, Tetsuya Ishikawa, Kenichi Masui

**Affiliations:** 1 Department of Anesthesiology, Showa University School of Medicine, 1-5-8 Hatanodai, Shinagawa, Tokyo, 142-8666 Japan; 2 Department of Intensive Care Medicine, Showa University School of Medicine, Tokyo, 142-8666 Japan; 3 Department of Obstetrics and Gynecology, Showa University School of Medicine, Tokyo, 142-8666 Japan

**Keywords:** Severe hyponatremia, Hysteroscopic surgery, Brain edema, Seizures, Neurological deficit, Electrolyte-free irrigation fluid

## Abstract

**Background:**

Hyponatremia can be developed during hysteroscopic surgery with electrolyte-free irrigation fluid. We experienced severe hyponatremia with postoperative seizures and confirmed mild brain edema.

**Case presentation:**

A quadragenarian female patient underwent a 2-h hysteroscopic myomectomy with electrolyte-free fluid for uterine distension under general anesthesia. Plasma sodium level of 84.1 mmol/L 100 min after the start of surgery indicated excessive absorption of the irrigation fluid. Acute severe hyponatremia was diagnosed with significant edema in the conjunctiva, lip, and extremities. She was treated with a continuous infusion of hypertonic saline. However, seizures and cerebral edema developed 7 h later. The patient recovered without neurological deficits at postoperative day 2.

**Conclusion:**

The electrolyte-free irrigation fluid can be absorbed rapidly during hysteroscopic surgery. Its interruption with hyponatremia should be considered against prolonged surgery. Especially under general anesthesia, caution should be exercised because the typical symptoms of hyponatremia such as nausea and confusion are blinded.

## Background

Hyponatremia is a severe complication of hysteroscopic myomectomy, caused by the absorption of fluid media used for uterine distension via damaged blood vessel and myometrium [1–7]. Absorption of an electrolyte-free fluid media can decrease the plasma colloid osmotic pressure, which results in fluid movement from intravascular to extravascular space. These changes can cause low arterial pressure, circulatory failure, cerebral edema, convulsions, and even death by osmotic demyelination [8]. Here, we report a case of severe hyponatremia developed during a hysteroscopic surgery with postoperative seizures and confirmed mild brain edema, and fully recovered on postoperative day 2.

## Case presentation

**Fig. 1:**
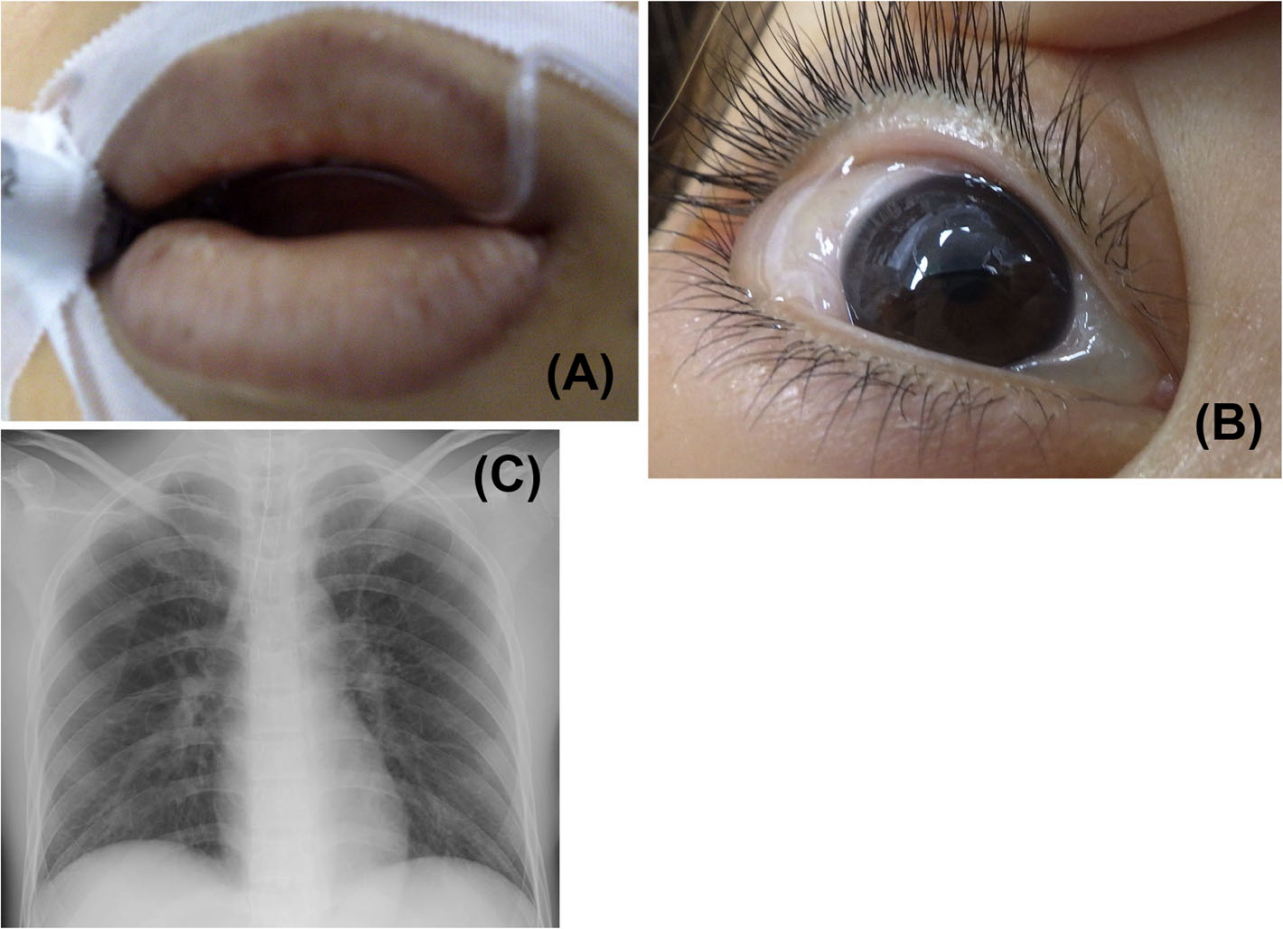
Edema of lips (**a**) and conjunctiva (**b**), and chest X-ray (**c**) at the end of the operation

A written patient consent was obtained for this report. A quadragenarian female patient (height 151 cm, body weight 37 kg) was scheduled for a hysteroscopic myomectomy under general anesthesia. Her chief complaint was hypermenorrhea. Preoperative ultrasound revealed a 3.6-cm submucous uterine myoma which protruded into the uterine cavity (protrusion ratio: 60–70%). Gonadotropin-releasing hormone therapy was initiated 3 months prior to the operation to improve anemia and shrink the myoma. Her preoperative laboratory tests were normal except the serum bilirubin level of 1.3 mg/dL. Electrocardiogram, non-invasive blood pressure, and pulse oximetry were applied for intra-anesthesia monitoring. Immediately before the anesthesia induction, the vital signs were as follows: blood pressure, 114/80 mmHg; heart rate, 82 bpm; and SpO_2_, 100% (FiO_2_ = 0.21). General anesthesia was induced with fentanyl, propofol, and rocuronium, followed by tracheal intubation and maintained with desflurane and remifentanil. The urinary catheter was not inserted as the estimated operation time was 1 h. Electrolyte-free 3% d-sorbitol solution (Uromatic S®, 165 mOsm/L, Baxter Limited, Tokyo, Japan) was used for monopolar electrosurgery. The patient was placed in a lithotomy position. The solution bag was placed at approximately 100 cm height from the surgical table without any additional pressure applied to the bag. The difference between the fluid inflow and outflow increased over time. During the latter half of the surgery, a substantial spillage was observed on the floor. A mild decrease in blood pressure was observed throughout the surgery. Approximately 100 min after the start of surgery, the plasma sodium level was estimated as 84.1 mmol/L. At the end of the 123-min surgery, the difference between the inflow (21 L) and the collected outflow (14 L) was 7 L excluding the spillage. At this time, the vital signs were as follows: blood pressure, 80/34 mmHg; heart rate, 60 bpm, SpO_2_, 99% (FiO_2_ = 0.45). Premature ventricular contractions were observed 5 to 10 times per minute. The intravenous fluid infused was 780 mL in total. The urinary catheter was inserted due to prolonged surgical time. Immediately, 1000 mL of urine was excreted. After the drapes were removed, distention of the lower abdomen and navel was noticed. No abdominal fluid wave on palpitation, and no findings of intraperitoneal fluid retention by an abdominal ultrasound exam were observed. However, there were significant edemas in the conjunctiva, lip, and fingers (Fig. 1). Arterial blood gas test revealed severe hyponatremia and acidemia (pH 7.188; plasma sodium level 88.1 mmol/L; partial pressure of carbon dioxide 41.7 mmHg; partial pressure of oxygen 147.7 mmHg). Chest X-ray suggested mild pulmonary edema (Fig. 1). With these findings, we diagnosed acute hyponatremia due to water intoxication. One hour after the immediate administration of 20 mg furosemide, an additional 1000 mL of urine was excreted. However, the second arterial blood gas test still indicated severe hyponatremia (plasma sodium level 90.8 mmol/L). She was admitted to the intensive care unit (ICU) under sedation and mechanical ventilation with tracheal intubation.

**Fig. 2:**
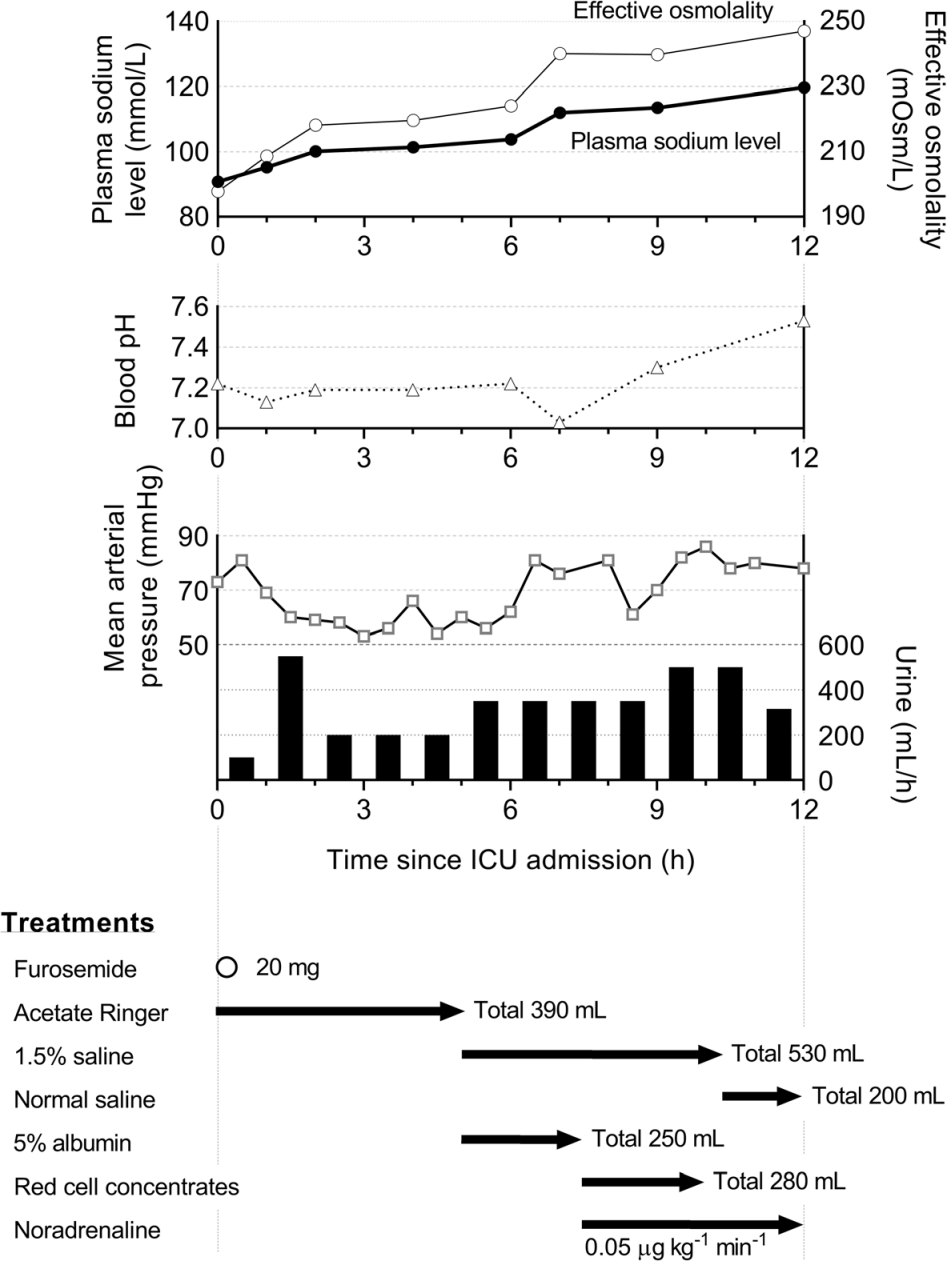
Time courses of plasma sodium level, effective osmolality, blood pH, mean arterial pressure, urine output, and treatments for hyponatremia in intensive care unit. Each symbol indicates each measurement and each black bar shows hourly urine output. For the treatments, each arrow indicates the duration of the treatment, and open circle indicates the time of furosemide administration

**Fig. 3:**
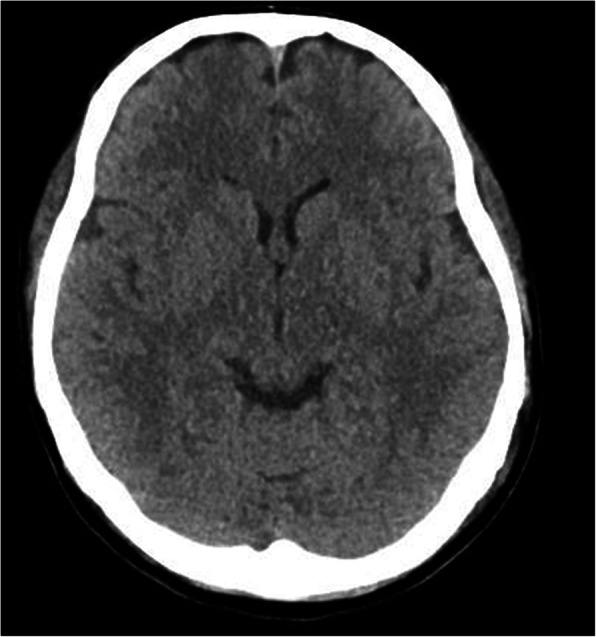
Head computed tomography immediately after the seizure attack. The image demonstrates mild cerebral edema 7 h after ICU admission

The details of correctional treatment, and transition of plasma sodium level and urine output are described in Fig. 2. Sedation was maintained with propofol. During the first 4 h, acetate Ringer solution and an additional 20 mg furosemide were given. Although the diuresis over 100 mL/h was maintained, metabolic acidosis, high lactate level, and low blood pressure persisted. After 6 h from the ICU admission, the coexistence of intravascular dehydration was diagnosed by increased lactic level and progression of metabolic acidosis. Accordingly, 5% albumin followed by blood transfusion was administered, and continuous infusion of noradrenaline was initiated at 0.05 μg/kg/min. For sodium correction, continuous administration of 1.5% saline was also initiated. The patient developed a few short seizures at 7 h after ICU admission and recovered spontaneously. A head computed tomography taken immediately after the attack showed mild cerebral edema (Fig. 3). At this time, the plasma sodium level was 112.0 mmol/L with metabolic acidosis. Treatments of 1.5% saline and noradrenaline infusion were continued until 12 h since the ICU admission. Three hours later (15 h after the ICU admission), the patient was extubated under light sedation with propofol after confirming the improvement of the electrolyte balance (plasma sodium level 125.6 mmol/L), blood pressure stabilized, and the frequency of premature ventricular contractions decreased. Glasgow coma scale was E3V1M4 immediately after extubation. A further 3 h later, no abnormalities were found in cerebral magnetic resonance imaging. At 7 h later after extubation, Glasgow coma scale was E3V5M6. On the second postoperative day, her consciousness fully recovered. She was released from the hospital on the sixth postoperative day, following a physical examination by a neurologist and a second cerebral magnetic resonance imaging, which revealed no neurological deficits.

## Discussion

In this case, severe hyponatremia occurred during a 2-h hysteroscopic myomectomy with electrolyte-free solution. Although a seizure attack and mild brain edema occurred, the patient fully recovered on the second postoperative day.

Hysteroscopic surgery is generally less invasive than laparoscopic or open surgery when the patient selection is appropriate. As this case had 3.6 cm leiomyoma, smaller than 4–5 cm, the indication was applicable [9]. However, excessive fluid absorption can be a life-threatening complication in a hysteroscopic surgery. A previous study examined the relationship between the type of submucous leiomyoma and fluid absorption volume [3]. Type 0, 1, or 2 myoma (no, < 50%, or ≥ 50% myometrial extension, respectively [9]) resulted in mean absorption volume of 450 (range: 0–1700), 957 (0–3000), or 1682 (50–4500) mL over 36, 47, or 56 min of the operation time, respectively. The result has shown a strong relationship between the absorption volume and the operation time. The interruption of the surgery should be considered with the assessment of the difference between the volume of inflow and outflow fluid [2]. In our case, an indication for the interruption was the operation time extended beyond 1 h with a large difference between inflow and outflow, although the estimation of the absorption volume was difficult because of the substantial spillage. A rapid termination of the operation is recommended after a fluid loss > 1500 mL, and the operation should be interrupted with a loss > 2000 mL [3]. Another criteria for interruption may be plasma sodium level < 120 mmol/L indicating severe hyponatremia [8].

Treatment of severe acute hyponatremia should be considered. A guideline proposes a treatment strategy for hyponatremia with severe symptoms (1D and 2D indicate strong and weak recommendations with low evidence) [10]. Briefly, intravenous infusion of 150 ml 3% hypertonic saline over 20 min (1D), and repeated infusion of the same solution twice until 5 mmol/L increase of Na, is observed in the first hour (2D). In a case without improvement of symptoms after 5 mmol/L increase, 3% hypertonic saline infusion or a continuous hypertonic saline infusion, aiming for 1 mmol/L increase per hour until the improvement of the symptoms, or increase of plasma sodium level 10 mmol/L in total, or recovery to Na 130 mmol/L (1D). In our case, plasma sodium level gradually increased since the ICU admission with a treatment of 1.5% saline started at 5 h after the admission, seizures, and mild brain edema developed at 7 h after the admission (Fig. 3). Sodium correction at a higher rate might have been better to avoid these symptoms. Overly rapid correction of hyponatremia can develop osmotic demyelination, where neurological findings improve in the early phase but are followed by a new progression of, sometimes permanent neurological deficits in one to several days later [11]. We treated hyponatremia with a mild increase in plasma sodium level and the patients fully recovered on postoperative day 2.

Electrolyte-free 3% d-sorbitol solution was used in this case for the monopolar electrical system. Nowadays, isotonic fluid is also used in hysteroscopic surgery to avoid dilutional hyponatremia. However, cases have been reported that isotonic fluid can result in severe hypokalemia and metabolic acidosis with arrhythmia [12] or severe hyperchloremic acidosis within 30 min into the surgery [13]. Regardless of the fluid type for uterine distension, fluid balance monitoring is principal in the anesthesia management. One limitation of this report is the lack of the detailed irrigation fluid balance.

As the significant edema and the difference between the fluid inflow and outflow indicated the absorption of irrigation fluid, we administered furosemide to excrete water from the body. It should be noted that chronic furosemide intake can develop hyponatremia [11, 14] due to the inhibition of sodium absorption in the kidney.

We experienced severe hyponatremia with a postoperative seizure attack and mild brain edema in a case undergoing a 2-h hysteroscopic procedure with electrolyte-free solution as irrigation fluid. During the surgery, it is essential to monitor the fluid balance closely, and the interruption of the surgery should be considered by prolonged surgery and a large difference between inflow and outflow fluid. Under general anesthesia, caution should be exercised because the typical symptoms of hyponatremia such as nausea and confusion are blinded. Hyponatremia was treated with hypertonic saline administration and left no permanent neurological deficits.

## Data Availability

Not applicable.

## Abbreviations

ICU: Intensive care unit
